# Extreme Hydro-Meteorological Events Influence to Water Quality of Small Rivers in Urban Area: A Case Study in Northeast Poland

**DOI:** 10.1038/s41598-020-67190-4

**Published:** 2020-06-24

**Authors:** Katarzyna Puczko, Elżbieta Jekatierynczuk-Rudczyk

**Affiliations:** 1 Department of Environmental Protection, Faculty of Biology, University of Bialystok, Ciołkowskiego 1 J, 15-245, Białystok, Poland; 2 Department of Environmental Protection, Faculty of Biology, University of Bialystok, Ciołkowskiego 1 J, 15-245, Białystok, Poland

**Keywords:** Hydrology, Environmental impact, Hydrology

## Abstract

This paper presents an impact of hydro-meteorological extreme events and urban catchment to water quality in small rivers in Białystok (Poland). The results from a five-year study have taken into account droughts, continuous precipitation, and storm precipitation causing flash floods. Extreme hydro-meteorological events has a different impact on the physical and chemical parameters of water. It was found that the largest change in water quality occurs on the 2nd day after the rainfall and changed concentration of some chemical parameters persists for a long time. The majority but, what’s important, not all of them are diluted after floods and concentrated after droughts. Flash flooding results in a large increase concentrations of DOC and selected forms of phosphorus. Higher values of EC, Eh, Mg^2+^, HCO_3_^-^, Cl^-^, SiO_3_^2-^, NO_3_^-^N, TN were observed during drought compared to the average values from 2014–2018. A high degree of naturalness of the river valley and increased water retention results in a decreased concentration of NH_4_^+^-N, DOC and phosphorus forms. The buffer zone plays an important role in limiting the inflow of pollutants and nutrients from the catchment area. That is why it is worth undertaking restoration of river valleys in urban areas.

## Introduction

A growing interest in extreme weather events is reflected in many papers examined rainfall, flooding, heat, and drought. The research encompass all continents, but less than 10% of studies were focused in urban areas. This is surprising because urbanized areas are most exposed to hydrometeorological extremes due to the high concentration of people and infrastructure^1^.

Extreme events are defined across six major disciplines that examine them (climatology, earth science, hydrology, ecology, engineering and social science)^1^. In our work, we focus on the definition, which is based on the assignment of any flow amplitude thresholds that identify floods and low flows in such a way that exceeding these thresholds is understood as the beginning of an extreme hydrological event. Floods resulting from storm precipitation refer to zone of high water levels. Floods resulting from continuous precipitation refer to above average multi-year flows. Continuous precipitation causes a slight increase in water levels in the river, but significantly affects its quality^2^. The analysis of no-rain periods concerns the zone of low water levels.

Extreme hydro-meteorological events, such as heavy precipitations or lack thereof, floods and droughts, are becoming more intense and frequent due to climate change and can strongly impact water quality^3^. Most studies focus on the amount of water, on whether it is too little or too much^4^, but there also numerous studies on the effects of climate extremes on water quality. It is common knowledge that urbanisation drastically affects changes in river hydro-morphology and water quality. The problem of extreme hydro-meteorological phenomena is intensifying in the area of changing climatic conditions^5^.

Urbanisation affects the change of the water cycle. Urban rivers are drastically modified when compared to agricultural and forested freshwater ecosystems^6^. Urban rivers often lose their hydraulic contact with their catchment. In natural ecosystems, surface water is connected with the hyporheic zone and groundwater. In urban rivers, this contact is disturbed or does not occur at all. Impermeable surfaces prevent infiltration of water, and rain drains quickly into a storm water sewage system^7^. Shortening the time of flow concentration increases its culmination and the volume of flood wave in the catchment, causing an increase of flood risk and water pollution^6^. The rapid inflow of pollution from the urbanized catchment can change the quality of the habitat in the riverbed and river valley.

Very dangerous for human beings and appearing with increasing frequency are flash floods^8^. They occur after very intense rainfall in a short time, most often during summer storms. Water quality can also change as a result of intense continuous rainfall lasting from several days to several dozens of days. This type of precipitation occurs mainly in summer and autumn, in Polish lowlands. With the onset of drought, there is a sequential decline in precipitation, runoff, soil moisture, groundwater levels and stream flow^9^. Water quality may deteriorate to critical values during periods of prolonged low-flow conditions^10^. Many physical and chemical parameters of water may exceed critical values negatively affecting the river ecosystem and biocenosis.

The impact of floods and droughts on water quality is essential, especially for urban rivers, which are strongly modified by human activity. Any change in the quality of water in a city can have very serious consequences, ranging from ecological disturbances in the river ecosystem (self-purification of water is much more difficult here) to exclusion from the use of recreational areas. The main objective of this research was to determine the synergistic impact of extreme hydro-meteorological events and urban catchment on water quality of small rivers. Many physical and chemical water quality parameters have been tested to holistically capture the variability of water quality and to thoroughly examine the relationships and processes occurring during extreme hydrological conditions. The aim of the study was to show how the physical and chemical parameters of water change after heavy precipitations, intense continuous rainfall and during drought.

Research hypotheses:

1. During floods river water components are diluted in urbanised areas.
  - flash floods cause rapid dilution of physical and chemical parameters of surface water,
  - after continuous precipitations, the dilution of physical and chemical parameters of surface water takes place with a delay in relation to the dilution after flash floods.
2. Low water levels in rivers cause concentration of dissolved mineral and organic components in water.
3. The varied degree of naturalness of urban river valleys affects the quality of water during hydrological extremes.

## Study area

### Hydrological characteristics

Białystok lies in the Podlasie province, in the north-eastern part of Poland. The city is situated in the Białystok Upland of the Podlaskie Plain. The region is characterised by high natural values, and is part of what is known as the Green Lungs of Poland. Near the city, there are many unique natural ensembles on a European scale include the Narew National Park (a swampy valley with moraine hills typical of a braided river) and the Knyszyn Forest Landscape Park (one of the best preserved forest complexes in Poland). Due to the occurrence of unique environmental values, Białystok was the first city in Poland in 1993 to be admitted to the Healthy Cities Network of the World Health Organisation.

**Figure 1:**
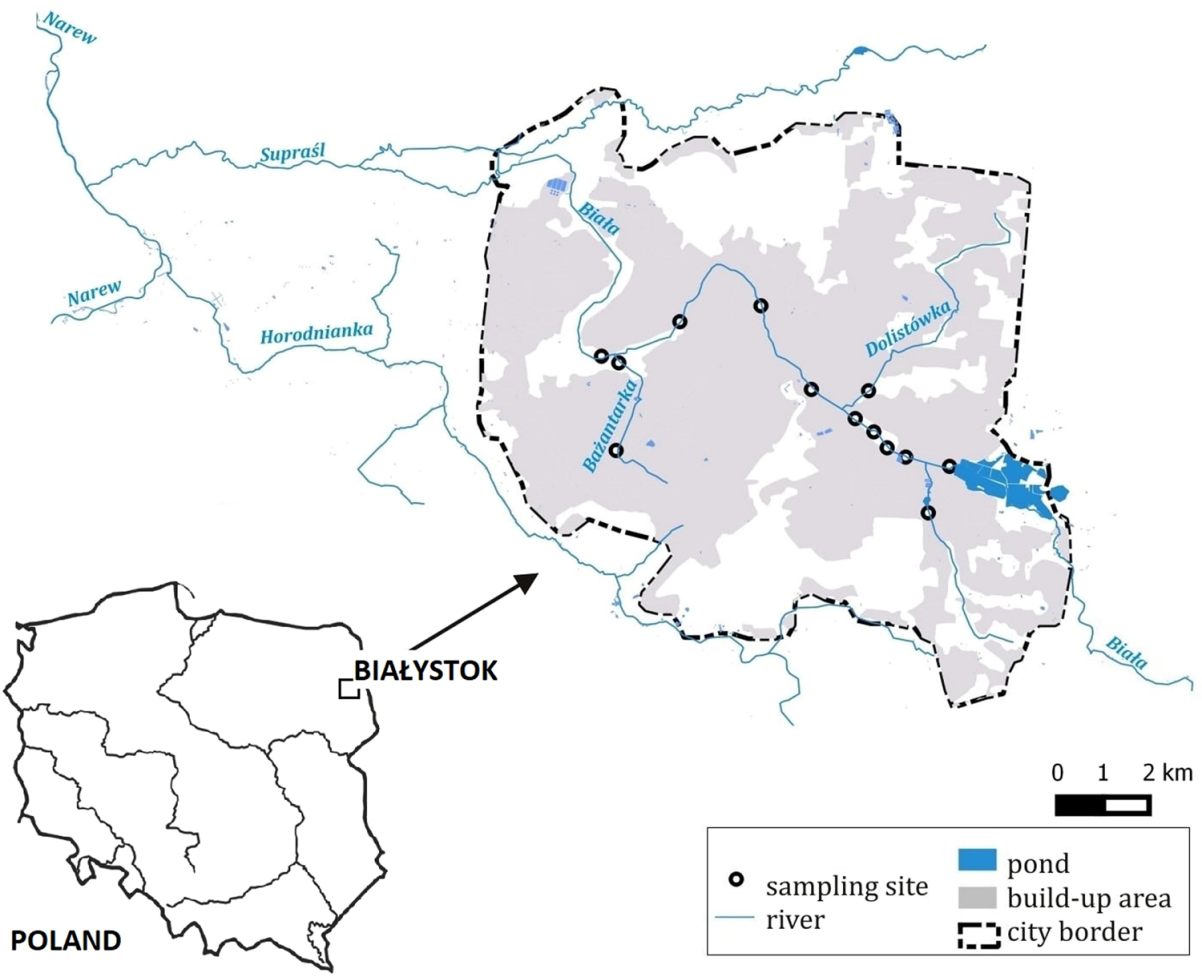
Map of sampling sites^50^.

In hydrological terms, the study area belongs to the Vistula river catchment in the Baltic Sea basin. Bialystok city is situated within the fourth-order catchment, including the Biała and Jaroszówka rivers and a fifth-order belonging entirely to the Biała catchment, including Dolistówka, Bażantarka and 15 small, temporary and unnamed watercourses^11^. (Fig. 1). The regime of the Białystok rivers is controlled by rainfall/snowfall-evapotranspiration, and mainly corresponds with the seasonal variation in net precipitation surplus. Natural water circulation in Białystok is strongly disturbed by anthropogenic activity, especially the rainwater sewage system. The drainage system cover 47.35% of the city^11^, which contributes to an unnatural shaping of the rivers’ flow rates and deterioration in water quality^12–14^.

**Figure 2:**
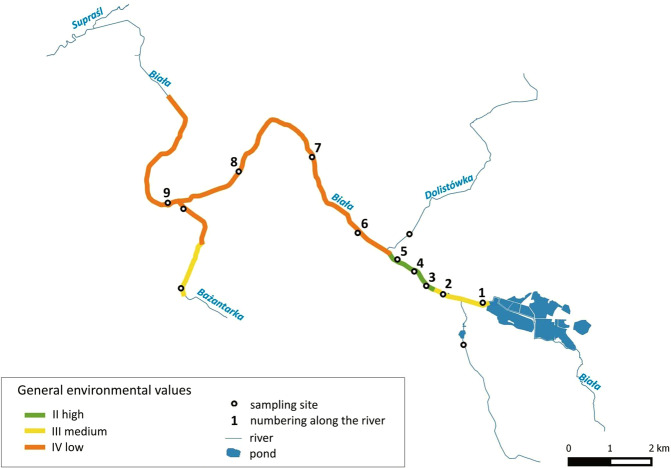
General nature and landscape valorisation of the Biała river valley and sampling sites^50^.

The length of the Biała river is 27.3 km, of which about 20 km is within the city of Białystok. The width of the river varies from 0.9 m to 6.0 m, and depths vary from 0.15 m to 1.5 m. Differences in river depth are caused by different bottom sediments, local regulation of the riverbed and damming up the river. Some sections of the Biała river are regulated, and the water flows in a straight line, with technically shaped banks. The natural river valley was changed by the elements of the communication infrastructure, drainage ditches and dikes. The average river flow is 1.2 m^3^/s. It greatly increases in the periods of thaws and heavy rains (high flow 5.97 m^3^/s), but during periods of non-precipitation, flow is significantly reduced (low flow 0.53 m^3^/s). Maximum flows with a probability of exceeding 10% and 1% is 12.4 m^3^/s and 23.0 m^3^/s, respectively^15^. In the upper course, the Biała river flows into a large depression of the melting point, of which the central part is fish ponds and the Dojlidy recreation reservoir in the suburbs of the city^16^. In the middle course, the river has hydraulic contact with two ponds with high trophy. In 2016, a river restoration was carried out on a section of about 1 km. The Biała river was meandrified, floodplains were restored, and swamp vegetation areas were created (Fig. 2).

The Dolistówka river is a right-bank tributary of the Biała river with a catchment of 16.2 km^2^, of which 80% is located in urban area (Fig. 1). The river is characterised by high flows of flood water and low flows in dry periods. The river is not monitored, and no hydrological measurements are carried out.

The Bażantarka river is the left tributary of the Biała river (Fig. 1). The Bażantarka catchment is located almost entirely within the city limits (99%). Bażantarka is the most anthropogenically transformed river in Białystok. Some sections of the river were directed to the rainwater drainage system. The river is characterised by a large water fall with an average of 5.5‰^11^. In the Bażantarka river valley, there are ponds (Marczukowskie Ponds) that have undergone restoration. Marczukowskie Ponds are one of the most attractive scraps of natural nature in Bialystok. They are characterised by the occurrence of fauna species characteristic of wetlands.

### Meteorological characteristics

Białystok has a continental climate, characterised by warm summers and long frosty winters^15^. The average annual air temperature is 6.8 °C, the warmest month is July (the average monthly temperature is 17.3 °C), and the coldest month is January (−4.3 °C). The average annual air temperature is 6.9 °C. The average annual sum of atmospheric precipitation in Bialystok from the years 1961–1995 amounted to 598 mm and from 1981–2010, amounted to 577 mm (Table 1). The highest rainfall occurs from May to August, with the maximum in July. The lowest rainfall occurs between January and March. Snowfall accounts for about 21% of the annual amount of precipitation. In Bialystok, there are heavy rains, and it rains, on average, 24 times a year. Most often, storm days are observed in May and June. Snow cover lasts an average of 96 days a year. It is observed from November to April, but it does not persist constantly due to the thaw^11,17^.

**Table 1:** The sums of precipitation in individual years at the meteorological station Białystok No. 353230295 (in 1961-1995^17^ and 1981-2010 - the average sum of monthly precipitation^18^).

Months	1961–1995	1981–2010	2014	2015	2016	2017	2018
January	34	33	44	45	27	13	27
February	27	27	24	31	56	45	15
March	33	33	31	41	58	51	23
April	37	34	21	29	37	78	41
May	60	64	66	103	47	101	31
June	71	69	82	26	44	116	22
July	76	75	69	63	187	83	145
August	69	66	60	5	69	108	26
September	57	56	21	34	22	123	66
October	45	41	4	36	136	113	40
November	46	41	26	81	55	48	21
December	42	38	53	61	53	55,3	78
Sum of							
the year	598	577	500	554	790	935	535

## Methodology

### Hydrological and meteorological data

The daily hydrological and meteorological data (from the years 2014–2017) were collected from the Institute of Meteorology and Water Management—National Research Institute (IMGW-PIB) database^18^. Data came from the weather station Białystok No. 353230295 and hydrological station Zawady No. 153230060. They were used to calculate the sum of annual precipitation, monthly precipitation sums and several days of precipitation sums, which were correlated with water quality features. Rainfall intensity and water level in the river was also used to determine periods of occurrence of heavy rains, continuous rains and dry periods. Correlations between precipitation and water quality were carried out in relation to various precipitation and rainfall periods.

### Water quality data

Water tests were conducted from 2014 to 2018 year. We collected water samples from rivers: Biała (9 sampling sites), Bażantarka (2 sampling sites), Dolistówka (1 sampling site) and one sample from smaller an unnamed watercourse (Fig. 1). Sampling sites were sampled on the same days. In 2014, samples were taken four times a year, in 2015, six times a year, in 2016, eight times a year, in 2017, ten times a year and in 2018, seven times a year. Samples were taken in all seasons of the year and at the time of the hydro-meteorological extremes.

In the field, a multi-parameter probe by HachLange was used to measure, at certain intervals, the temperature, electrolytic conductivity (EC), dissolved oxygen concentration, oxygen saturation of water (SW), reaction (pH) and redox potential (Eh).

Chemical water analyses were carried out in the laboratory of the Department of Environmental Protection, University in Białystok in accordance with ISO standard methods^19^. The following analyses were performed: total hardness, bicarbonates (HCO_3_^-^), calcium (Ca^2+^), sodium (Na^+^), potassium (K^+^), chlorides (Cl^-^) and silicon (SiO_3_^2-^). Magnesium concentration (Mg^2+^) was calculated as the difference between total hardness and calcium concentration.

Nutrient analysis included the following methods: ammonium nitrogen (NH_4_^+^-N) (indophenol blue colorimetry method), nitrate nitrogen (NO_3_^-^N) (by reduction and colour development with sulphanilamide and N-(1-Naphthyl)-ethylenediamine dihydrochloride) and nitrite nitrogen (NO_2_^-^N) (sulphanilamide method)^19^. Total nitrogen was analysed by Tecator 2300 (Kjeldahl analyser). Mineral nitrogen (TIN) was calculated as the sum of NH_4_^+^-N, NO_3_^-^N and NO_2_^-^N. Organic nitrogen (TON) was calculated from the difference between total nitrogen and ammonia. Total nitrogen (TN) was calculated as the sum of organic and mineral nitrogen. Phosphorus ions were determined by the molybdenum method measured according to standard methods^19^. Five phosphorus fractions were distinguished: total fraction (TP), dissolved fraction (DP), soluble reactive fraction (SRP), dissolved organic fraction (DOP) and particle fraction (PP). The total fraction was determined in non-filtered water after mineralisation. The soluble fraction was determined in water filtered through a filter GF/F with a pore diameter of 0.45 μm and mineralisation. The reactive fraction was determined in water filtered through a filter GF/F without prior mineralisation. Dissolved organic carbon concentration (DOC) was determined by Shimadzu TOC-5050A analyser with an IR detector. Concentrations of the total iron (TFe) were analysed by applying the spectrophotometric method with 1,10-phenantroline in unfiltered water samples after UV digestion with concentrated sulphuric (VI) acid and 30% hydrogen peroxide.

All collected results were used for the general characteristics of water quality in the rivers of Bialystok, and included the following parameters: EC, Eh, SW, Ca^2+^, Mg^2+^, HCO_3_^-^, Cl^-^, SiO_3_^2-^, TFe, K^+^, Na^+^, NH_4_^+^-N, NO_3_^-^N, NO_2_^-^N, TON, TN, DP, SRP, DOP, PP, TP and DOC. To explain the impact of hydro-meteorological extremes events, the same parameters were taken into account, and the analyses were used, that were performed during the events.

The work uses the results of environmental valorisation of the Biała river valley made by Tyszewski and Kardel^15^ (Fig. 2). Valorisation was carried out using the index method^20^. In the index method, four zones, subjected to separate valorisation (riverbed, coastal zone, zone of terraces and slopes, and the area of river valley) are separated, and the result (after taking into account weights) is summed up. The result is compared to a five-grade scale of environmental values (1—very high values, 5—very low values). The natural values of the Biała from each of the 14 sections of the river were assessed, taking into account the Bażantarka river as No. 12^15^ (Fig. 2). In our research, sampling locations along the Biała River were taken into account to show the impact of the environmental naturalness and the river restoration on water quality.

### Statistical analysis

The water quality characteristics assigned to different rivers in Białystok were compared among sampling sites using analysis of variance (ANOVA), and Tukey´s HSD (Honestly Significant Difference) test was carried out to determine in which sites significant differences occurred. Spearman’s rank correlation analysis was used to show the relationships among water quality variables, precipitations, daily water flow and the environmental valorisation. The statistical significance level was set at *p* = 0.05.

Principal component analysis (PCA) was used to visually examine and compare differences in water quality in periods of hydro-meteorological extremes phenomena in rivers not covered by hydrological monitoring. Due to the large number of parameters analysed, bearing in mind the readability of the charts, macronutrient and nutrients analyses are presented separately. PCA is a multivariate statistical method which is applied in environmental studies to explain data structures^21^. The following parameters were included in the PCA analysis: EC, Eh, SW, Ca^2+^, Mg^2+^, HCO_3_^-^, Cl^-^, SiO_3_^2-^, TFe, K^+^, Na^+^, NH_4_^+^-N, NO_3_^-^N, NO_2_^-^N, TON, TN, DP, SRP, DOP, PP, TP and DOC.

## Results

### Meteorological and hydrological conditions

**Figure 3:**
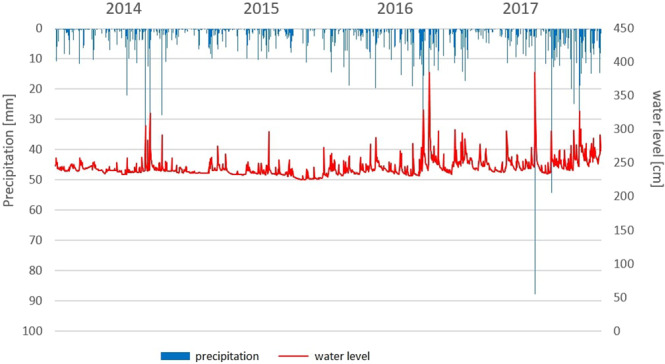
Daily flows in the Biała river in years 2014–2017.

Annual rainfall amounts for the analysed years have been presented in Table 1. In years 2014, 2015 and 2018, the sums of precipitation were lower, and in 2016 and 2017, they were higher compared to the average of years 1961–1995 and years 1981–2010. Months with the smallest sum of rainfall were in October 2014 and August 2015, with a value not exceeding 10 mm. Months with the highest sum of rainfall exceeding 110 mm were in July 2016 and 2018, June 2017, September 2017 and October 2016 and 2017. Maximum rainfall with the value 187 mm and continuous character was recorded in July 2016. The heavy rains were recorded in May and July 2017 year. The maximum one day precipitation was recorded on the 7th of May with the value 88 mm (nine hours of rainfall) (Fig. 3).

The analysis of flows in the Biała river in years 2014-2017 have been presented in Fig. 3. The years 2014 and 2015 were characterised by low dynamics of water levels and water flow intensity. In 2015, the lowest water levels and the lowest flows were observed. The years 2016 and 2017 were characterised by greater dynamics and occurrence of extreme flows and water levels resulting from sudden meteorological phenomena.

The water sampling dates coincided with the occurrence of hydrological extremes. Low water levels and low flow were captured in June 2015 (water level: 223 cm; water flow: 0.23 m^3^s^−1^) and August 2015 (water level: 225 cm; water flow: 0.15 m^3^s^−1^). The water samples were collected after a flash flood, in May 2017. The heavy rainfall caused a water level rise to 385 cm, and the flow rate reached a value of 12 m^3^s^−1^. The continuous rain in August 2016 resulted in an increase of the water level to 267 cm and a flow of 1.3 m^3^s^−1^.

### Water quality

The water quality parameters varied greatly in space. Statistically significant differences between general water quality parameters in rivers - the Biała, Bażantarka, Dolistówka and an unnamed stream - were identified for EC, Ca^2+^, Mg^2+^, HCO_3_^-^, Cl^-^, Na^+^ and K^+^ (Table 2). The highest mean value of EC (1,062 µS cm^−1^) was observed in the Bażantarka river, and the lowest mean value was observed in the Biała river. In the Dolistówka river, the highest mean value of chlorides was observed. The Bażantarka is a river with the most transformed physical and chemical parameters of water with elevated values of most macronutrients. The lowest mean values of macronutrients were observed in the unnamed watercourse with the exception of bicarbonates with a mean value of 68 mg C L^−3^ (Table 2).

**Table 2:** Water quality characteristics of rivers in Białystok in 2014–2018 (mean value).

Parameter		Biała	Bażantarka	Dolistówka	unnamed stream	Statistical significance of differences*
		*n* = 133	*n* = 40	*n* = 19	*n* = 22	
		**1**	**2**	**3**	**4**	
temp.		12.5	12.0	11.3	10.7	—
pH		7.86	7.92	7.75	7.80	—
EC	µS cm^−1^	662	1062	963	673	*1–2; 1–3; 2–4; 3–4*
Eh	mV	98.0	70.6	78.1	68.6	*—*
Oxygen	mg L^−3^	9.1	9.4	9.1	9.9	*—*
SW	%	85	88	80	89	*—*
Ca^2+^	mg L^−3^	70.9	120.2	107.7	101.2	*1–2; 1–3; 1–4*
Mg^2+^	mg L^−3^	15.8	26.5	24.8	22.5	*1–2*
HCO_3_^-^	mg C L^−3^	55.4	74.1	62.6	67.8	*1–2; 1–4*
Cl^-^	mg L^−3^	41.1	53.0	57.9	33.6	*1–2; 1–3; 2–4; 3–4*
SiO_3_^2-^	mg L^−3^	1.50	2.06	1.61	1.50	*—*
TFe	mg L^−3^	2.16	1.72	0.58	0.75	*1–3; 1–4*
K^+^	mg L^−3^	7.5	13.6	13.8	10.0	*1–2; 1–3; 2–4*
Na^+^	mg L^−3^	38.5	61.8	60.1	35.5	*1–2; 1–3; 2–4*
NH_4_^+^-N	mg L^−3^	0.365	0.306	0.712	0.251	*1–3; 2–3; 3–4*
NO_3_^—^N	mg L^−3^	0.882	1.958	2.085	1.103	*1–2; 1–3; 2–4; 3–4*
NO_2_^—^N	mg L^−3^	0.008	0.011	0.024	0.006	*1–3; 2–3; 3–4*
TON	mg L^−3^	2.807	6.440	5.157	3.603	*1–2; 1–3; 2–4*
TN	mg L^−3^	3.812	8.653	8.241	5.177	*1–2; 1–3; 2–4*
DP	mg L^−3^	0.095	0.096	0.122	0.091	*—*
SRP	mg L^−3^	0.045	0.061	0.081	0.055	*1–3*
DOP	mg L^−3^	0.050	0.035	0.040	0.037	*—*
PP	mg L^−3^	0.130	0.105	0.120	0.117	*—*
TP	mg L^−3^	0.217	0.200	0.242	0.208	*—*
DOC	mg L^−3^	9.1	8.9	8.3	8.9	*—*

*Tukey (HSD)/Analysis of the differences between the categories with a confidence interval of 95%.

Rivers in Białystok differed significantly in concentrations of organic and inorganic nitrogen forms (Table 2). The DOC concentration in all rivers was about 9 mg L^−3^. No significant differences were observed in the case of phosphorus compounds. The SRP concentration significantly differed in the Biała and Dolistówka rivers. The highest average concentrations of TP, SRP and DP were recorded in the Dolistówka river (Table 2). The concentration of TP in rivers was about 0.2 mg L^−3^ (Table 2).

### Hydro-meteorological events—water quality linkages

The results from statistical analyses indicated that the amount of rainfall significantly affects the quality of river water in Białystok. Daily water level and water flow also affect the physical and chemical parameters of water (Table 3). The water quality changes are the highest on the second day after the occurrence of the precipitation. The parameters of which the reaction to precipitation is the highest are EC, oxygen concentration, SW, Mg^2+^, HCO_3_^-^, Na^+^, K^+^ and TN. Mainly negative correlations with precipitation and water quality were observed. The exceptions with positive correlations were Eh and some phosphorous forms (DOP, TP).

**Table 3:** Results of the Spearman’s rank correlation analysis of water quality variables and hydro-meteorological conditions in Białystok.

Parameter	daily rainfall	2 days after rainfall	5 days after rainfall	30 days after rainfall	daily water flow
temp.	**−0.168**	0.018	−0.065	0.035	−0.084
pH	−0.096	−0.015	0.009	0.030	**−0.330**
EC	**−0.257**	**−0.336**	**−0.353**	**−0.342**	**−0.327**
Eh	**0.252**	**0.232**	**0.276**	**0.118**	**0.450**
Oxygen	**−0.211**	**−0.429**	**−0.305**	**−0.125**	0.080
SW	**−0.316**	**−0.379**	**−0.311**	0.014	0.031
Ca^2+^	0.048	**−0.180**	**−0.197**	**−0.154**	**−0.316**
Mg^2+^	−0.082	**−0.299**	**−0.326**	**−0.170**	0.062
HCO_3_^-^	**−0.153**	**−0.291**	**−0.363**	**−0.261**	**−0.311**
Cl^-^	**−0.217**	**−0.139**	**−0.194**	**−0.333**	**−0.333**
SiO_3_^2-^	−0.074	−0.080	**−0.199**	−0.021	**−0.329**
TFe	−0.029	0.081	0.097	**0.191**	**−0.217**
K^+^	−0.093	**−0.200**	**−0.236**	**−0.277**	**−0.151**
Na^+^	**−0.205**	**−0.207**	**−0.226**	**−0.231**	**−0.230**
NH_4_^+^-N	−0.057	−0.086	−0.080	**−0.206**	−0.048
NO_3_^–^N	0.025	−0.066	−0.087	**−0.208**	**−0.193**
NO_2_^–^N	**−0.197**	**−0.165**	**−0.115**	**0.132**	−0.067
TON	**−0.124**	**−0.139**	−0.045	−0.104	**0.162**
TN	**−0.188**	**−0.279**	**−0.272**	**−0.209**	0.094
DP	0.063	0.103	**0.125**	−0.014	**0.126**
SRP	−0.026	−0.068	−0.045	**−0.120**	**0.154**
DOP	0.092	**0.196**	**0.186**	0.100	0.022
PP	0.028	**0.140**	0.066	**−0.156**	−0.081
TP	0.072	**0.189**	**0.134**	−0.104	0.001
DOC	**0.142**	**0.111**	0.053	**0.230**	**0.221**

Values in bold are different from 0 with a significance level alpha = 0.05.

**Figure 4:**
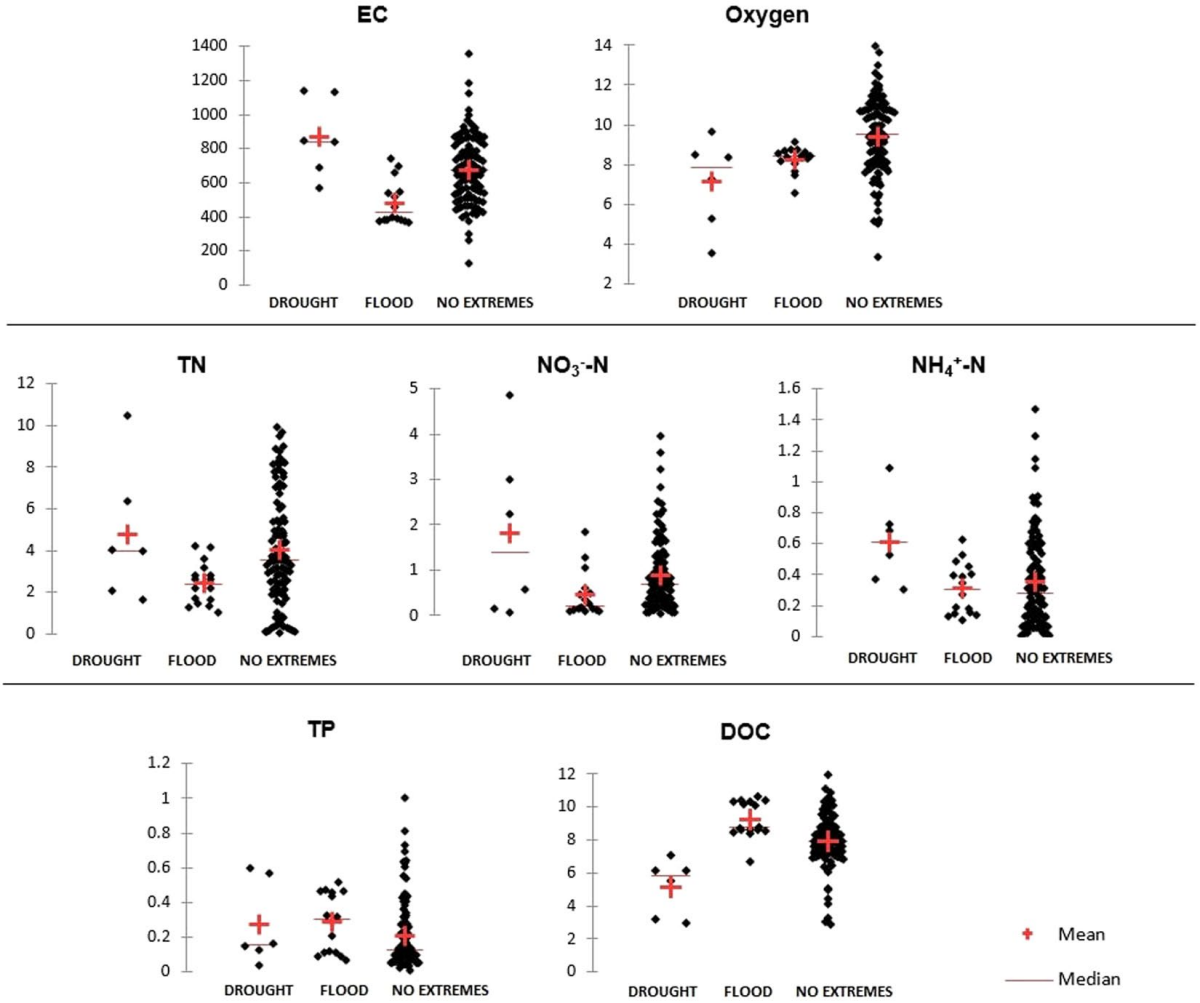
Scattergrams of physical and chemical parameters of water quality of the Biała catchment in the no extremes period and during droughts and floods.

Water quality results during drought and floods were presented for the B13 sampling site. This site was chosen because it is located in the final profile of the Biała river catchment. During drought, when there were low water flows and low water levels, most of physical and chemical water parameters of the Biała catchment were characterised by higher values compared to mean values from years 2014–2018 (EC, Eh, Mg^2+^, HCO_3_^-^, Cl^-^, SiO_3_^2-^, NO_3_^-^N, TN). The value of the EC increased above 1,000 µS cm^−1^ (Fig. 4). The magnesium concentration increased almost fourfold and concentration of nitrate nitrogen increased double during drought (Table 4). The concentration of TFe, DOC and most of the phosphorus forms (DP, DOP, PP, and TP) decreased. The DOC concentration decreased to 2.9 mg L^−3^ in June 2015 and to 5.5 mg L^−3^ in August 2015. No clear trends were found for Ca^2+^, NH_4_^+^-N, NO_2_^-^N, TON and SRP (Table 4).

**Table 4:** The water quality characteristics of rivers in Białystok in extreme hydro-meteorological periods, ↑ - parameter value increase, ↓ - parameter value decrease.

Parameter		B13 sampling site (n = 33)	Flash flood		Flood		Drought			
		*2014–2018*	*09.05.2017*		*02.08.2016*		*29.06.2015*		*25.08.2015*	
temp.		12.4	16.2		19.7		13.1		17.4	
pH		7.80	7.68	**↓**	7.92		8.18	**↑**	8.33	**↑**
EC	µS cm^−1^	844	370	**↓**	905	**↑**	1134	**↑**	1139	**↑**
Eh	mV	84.4	113.8	**↑**	88.0		108.6	**↑**	113.4	**↑**
Oxygen	mg L^−3^	9.3	8.0	**↓**	7.8	**↓**	9.7		8.5	
SW	%	86	77	**↓**	86		94	**↑**	90	**↑**
Ca^2+^	mg L^−3^	90.2	24.1	**↓**	27.1	**↓**	63.1	**↓**	154.7	**↑**
Mg^2+^	mg L^−3^	20.7	1.1	**↓**	3.7	**↓**	80.7	**↑**	70.4	**↑**
HCO_3_^-^	mg C L^−3^	62.9	33.7	**↓**	59.4	**↓**	79.9	**↑**	82.7	**↑**
Cl^-^	mg L^−3^	49.8	21.9	**↓**	16.6	**↓**	64.1	**↑**	76.6	**↑**
SiO_3_^2-^	mg L^−3^	1.72	0.7	**↓**	4.6	**↑**	2.0	**↑**	4.7	**↑**
TFe	mg L^−3^	1.6	1.2	**↓**	4.4	**↑**	1.1	**↓**	0.3	**↓**
K^+^	mg L^−3^	10.5	5.4	**↓**	10.1	**↓**	no data		no data	
Na^+^	mg L^−3^	51.0	19.6	**↓**	46.1	**↓**	no data		no data	
NH_4_^+^-N	mg L^−3^	0.376	0.178	**↓**	0.460	**↑**	0.302	**↓**	0.724	**↑**
NO_3_^–^N	mg L^−3^	1.348	0.168	**↓**	1.109	**↓**	4.849	**↑**	2.992	**↑**
NO_2_^–^N	mg L^−3^	0.009	0.004	**↓**	0.003	**↓**	0.004	**↓**	0.015	**↑**
TON	mg L^−3^	3.736	3.783		7.205	**↑**	5.265	**↑**	2.644	**↓**
TN	mg L^−3^	5.4	4.1	**↓**	2.8	**↓**	10.4	**↑**	6.4	**↑**
DP	mg L^−3^	0.107	0.103		0.049	**↓**	0.051	**↓**	0.005	**↓**
SRP	mg L^−3^	0.046	0.030	**↓**	0.012	**↓**	0.047		0.002	**↓**
DOP	mg L^−3^	0.061	0.073	**↑**	0.037	**↓**	0.003	**↓**	0.003	**↓**
PP	mg L^−3^	0.110	0.225	**↑**	0.014	**↓**	0.096	**↓**	0.033	**↓**
TP	mg L^−3^	0.217	0.328	**↑**	0.110	**↓**	0.147	**↓**	0.038	**↓**
DOC	mg L^−3^	8.6	10.3	**↑**	6.7	**↓**	2.9	**↓**	5.5	**↓**

During a flash flood in May 2017, all physical and chemical parameters of water were characterised with lower values compared to mean values from years 2014–2018. The value of the EC decreased to 370 µS cm^−1^. Concentrations of HCO_3_^-^, Cl^-^, SiO_3_^2-^, Na^+^ and K^+^ decreased twice (Table 4). The flash flood resulted in a decrease of nitrogen forms and an increase of DOC and phosphorous forms concentrations in river water. The PP concentration increased twice, and the nitrate nitrogen concentration decreased to value of 0.2 mg L^−3^ (Table 4). Continuous rainfall in July 2017 caused a slight increase in the EC value to 905 µS cm^−1^. The concentration of TFe and SiO_3_^2-^ increased almost three-fold. Concentrations of other physical and chemical parameters of water were lower compared to the mean values from years 2014–2018 (Table 4). The concentrations of phosphorous forms decreased twice after continuous rainfall, and the DOC concentration decreased to a value of 6.7 mg L^−3^. TON and ammonium nitrogen response decrease in concentration in river water (Table 4).

Both droughts and floods resulted in decreased oxygen concentration. Drought mostly increased nitrogen forms concentration, which is in contrast to floods, where nitrogen forms concentrations decreased. In the case of DOC, the situation was the opposite; during drought, the concentration decreased, and during flood, the concentration increased (Fig. 4).

**Figure 5:**
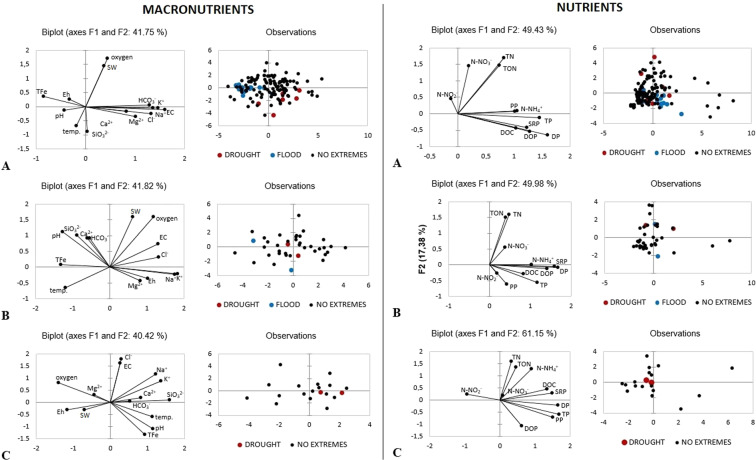
Principal component analysis (PCA) biplot (**A**) Biała river; (**B**) Bażantarka river; (**C**) Dolistówka river) show water sampling sites (characterised by flood, drought, no extreme) and factor loadings of physical and chemical parameters of water.

PCA analysis revealed distinctions between water quality parameters in samples from river water in a hydro-meteorological extremes period and in river samples collected in a no-extremes period in Białystok (Fig. 5). Table 5 presents the variability values of F1 and F2, as well as eigenvectors of general water quality parameters and nutrients in rivers: the Biała, Bażantarka and Dolistówka. The biplots and eigenvectors of PCA analyses for general chemical parameters of water differ in the studied rivers. The first component explains less than 30% and the second less than 20% of the total variance. Samples from the flood period are shown mostly within the left side of biplot, while in the right side, there are samples from the drought period. The first component referring to nutrients, which explains more than 29% of the total variance within the dataset, is characterised by positive loadings for phosphorous forms, DOC and nitrogen forms with the exception of nitrite nitrogen. The results of PCA analysis for nutrients are similar in all rivers studied. PCA biplots show that phosphorus forms and DOC had the greater impact to water quality during floods and nitrogen forms during droughts (Fig. 5).

**Table 5:** Eigenvectors of PCA analysis.

Variability (%)	Biała		Bażantarka		Dolistówka	
	F1	F2	F1	F2	F1	F2
	27.6	14.1	27.2	14.7	22.0	18.4
temp.	−0.053	−0.261	−0.270	−0.200	0.298	−0.173
pH	−0.119	−0.047	−0.289	0.344	0.299	−0.313
EC	0.443	−0.041	0.293	0.226	0.068	0.462
Eh	−0.089	0.099	0.231	−0.113	−0.307	−0.086
Oxygen	0.121	0.661	0.265	0.492	−0.367	0.233
SW	0.103	0.558	0.141	0.492	−0.186	−0.089
Ca^2+^	0.229	−0.065	−0.137	0.283	0.215	0.057
Mg^2+^	0.280	−0.134	0.186	−0.135	−0.114	0.091
HCO_3_^-^	0.358	0.024	−0.124	0.285	0.138	0.010
Cl^-^	0.367	−0.100	0.296	0.098	0.076	0.509
SiO_3_^2-^	0.009	−0.344	−0.202	0.310	0.420	0.028
TFe	−0.235	0.138	−0.299	0.025	0.246	−0.383
K^+^	0.377	−0.021	0.415	−0.063	0.360	0.250
Na^+^	0.404	−0.015	0.394	−0.072	0.321	0.332
Variability(%)	29.3	20.1	32.6	17.4	38.4	22.7
NH_4_^+^-N	0.289	0.024	0.308	0.005	0.239	0.439
NO_3_^-^N	0.057	0.499	0.113	0.228	0.021	0.067
NO_2_^-^N	−0.036	0.155	0.054	−0.106	−0.246	0.085
TON	0.211	0.503	0.119	0.613	0.120	0.462
TN	0.234	0.586	0.145	0.654	0.087	0.550
DP	0.456	−0.220	0.501	−0.034	0.439	−0.075
SRP	0.349	−0.147	0.475	−0.021	0.395	0.101
DOP	0.365	−0.187	0.421	−0.049	0.163	−0.368
PP	0.301	0.032	0.128	−0.248	0.398	−0.246
TP	0.415	−0.045	0.350	−0.232	0.447	−0.208
DOC	0.295	−0.148	0.247	−0.119	0.356	0.156

### Naturalness of the environment vs extreme phenomena

Spearman’s rank correlation analysis indicated that general environmental values of the Biała river valley were significantly negatively correlated with EC, HCO_3_^-^, Cl^-^, K^+^, Na^+^ and all the nitrogen forms with the exception of organic nitrogen. Significant positive correlation was observed between the environmental valorisation value and DOC and TFe (Table 6).

**Table 6:** Results of the Spearman’s rank correlation analysis of water quality variables and environmental valorization of the Biała river valley^15^.

Parameter	Environmental valorization value
temp.	0.013
pH	0.029
EC	**−0.319**
Eh	0.106
Oxygen	0.067
SW	0.093
Ca^2+^	−0.040
Mg^2+^	−0.085
HCO_3_^-^	**−0.191**
Cl^-^	**−0.244**
SiO_3_^2-^	−0.117
TFe	**0.150**
K^+^	**−0.402**
Na^+^	**−0.396**
NH_4_^+^-N	**−0.173**
NO_3_^-^N	**−0.312**
NO_2_^-^N	**−0.187**
TON	−0.126
TN	**−0.227**
DP	−0.029
SRP	−0.128
DOP	0.135
PP	0.110
TP	0.063
DOC	**0.173**

Values in bold are different from 0 with a significance level alpha = 0.05.

**Figure 6:**
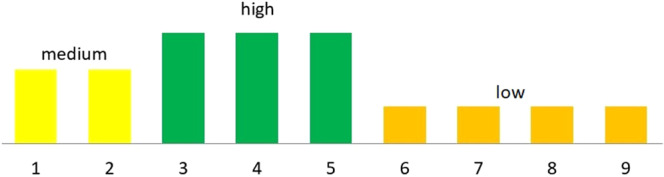
Degree of naturalness of the studied sections of the Biała river valley (1–9 sampling sites (Fig. 1)).

**Figure 7:**
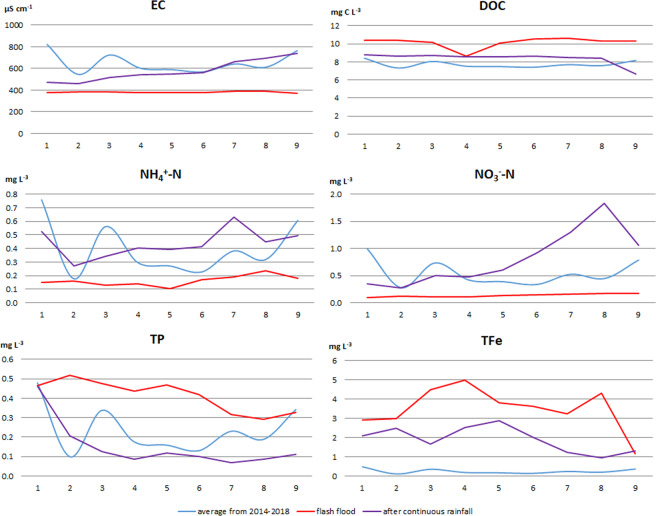
Concentrations of physical and chemical water parameters of the Biała river valley (1–9 sampling sites (Fig. 1)) during extreme hydro-meteorological events compare to average values from 2014–2018.

The environmental values of the sampling sites are presented in Fig. 6. Extreme hydro-meteorological events affected water components in different way. Flash flood results decreased the EC, and its values were similar in all sampling sites compared to average values from 2014–2018 (Fig. 7). After continuous rainfall, the EC values increased along the river. The river sections with a high degree of naturalness of the environment were characterised by reduced concentrations of TN, TP and DOC. Flash floods and continuous precipitation have an influence on and increase the DOC compared to average values from 2014–2018. Concentrations of nitrate nitrogen ions decrease after flash flooding compared to the period with no extremes. Continuous precipitation effects the increase of nitrogen forms concentration. We observed an increase in TP concentration after heavy rainfall. The concentration of TP in the period of continuous precipitation was lower than the average from years 2014–2018. Both hydro-meteorological extremes phenomena resulted in an increase of TFe concentrations (this concerns the central part of the revitalized section of Biała river) (Fig. 7).

## Discussion

Climate change, considered on a local scale, is particularly evident in urban areas^22^. The unnatural surface coverage of urban areas generates an “urban heat island”, which increases convection processes in the atmosphere and creates storm clouds, and storm precipitation that cause flash floods^23^. The characteristics of the variability of climate indicators for Białystok, developed using data from the period 1981–2015, indicate a statistically significant increase in the value of maximum precipitation. In addition, a growing trend was noted for the average duration of low water levels and the length of the longest period without precipitation^24^. Floods and droughts occurred in the short five-year research period, which confirms that climate change affects the intensity of extremes. The flash flood in May 2017 caused paralysis of the greater part of the city^25^. Climate change manifested by increase in the frequency and intensity of extreme hydro-meteorological events has a direct impact on the physical and chemical parameters of surface water^26,27^. Natural processes occurring in waters are disturbed, and the quality of river water is deteriorating. Rainwater is contaminated again in storm water drainage systems and then flows into rivers.

Most river valleys in Białystok were engineering modified, and the water flow was disturbed. The quality of surface water has been significantly transformed^12^. However, the rivers still play an important role in the city. They soften some elements of the urban climate (amplitude of air temperature, humidity). River valleys decide about the biodiversity of the area and have a recreational function for residents. Therefore, more and more work is being done to preserve their naturalness through a restoration programs.

Our research indicates that, hydro-meteorological extremes influence river water quality. Depending on whether there is continuous or storm precipitation, we observe changes in concentrations of many chemical parameters of water. Nutrients undergo various chemical transformations and processes and depend on the rainfall conditions, physical nature of the river and its catchment, and the land cover^28^. It was found a slightly different effect of the nature of precipitation on the quality of surface waters. Water quality analysis carried out on a day after a heavy rainfall indicated that most of water components were diluted and their concentrations decreased. The exceptions are DOC and phosphorus compounds, of which the concentration increased. Increased DOC levels after rainfall have also been shown in studies by Gao *et al*^29^. and Delpla *et al*.^30^. In general, surface water in the Białystok region contain large amounts of organic carbon because of the numerous areas of peat in the Biała river valley^31^. Surface runoff causes leaching and transport of carbon compounds, which results in an increase of DOC concentration^32,33^. An understanding of dissolved organic matter (DOM) transformation processes in urban areas is limited. During baseflow, DOM mainly comprises material from algal biomass. During the flood, other sources of organic matter are activated, and the DOM stores may be re‐connected to the main channel. Algal biomass and biofilms are rapidly flushed from the storm drainage-sewerage and mineralized^34^. The mineralization processes influence an increase in the concentration of phosphorus forms, which are hardly incorporated into biomass during flash floods.

Continuous precipitation results in dilution of chemical water parameters due to the water volume transported during rainfall event. They cause immediate change of physical river water parameters such as temperature, EC, dissolved oxygen concentration, SW and Eh. Some water components such as sodium, bicarbonate, chloride, TON and DOC respond to precipitation immediately and are diluted. The largest change in water quality occurs on the 2nd day after rainfall (Table 3). The extended time of hydrochemical changes indicates anthropogenic pressures of the area^35^. If the natural environment were changed to a small extent, one would expect a large initial load and a significant reduction in the subsequent hours of continuous precipitation. The shift to the next day indicates the continuous possibility of chemical leaching from the catchment. For some of the tested chemical water parameters, the process remains at a statistically significant level for many days, indicating a significant transformation of the environment as a result of the urbanization process. This applies to both types of precipitation (continuous and storm precipitation). According to Kozak *et al*.^28^, our studies indicates that some water quality parameters are independent of the rainfall influence.

Drought often causes subsequent hydrological effects such as reduced catchment runoff, small river flows and changes in water quality^36^. Our studies show that drought in urban areas results in increases of EC values and concentrations of most of the macronutrients. Low water level and limited precipitations result in decreased concentrations of organic carbon and phosphorous forms. A high increase in content of nitrate ions was observed. In general, many studies confirm that mixed drought responses from different nutrients are relatively common^37–39^. Dissolved nutrients showed little change, but TP increased in small natural rivers^33^. Point source discharges likely contributed to increased nitrate concentrations in urban catchments^40^. Some studies link higher nitrate concentrations to reduced dilution of groundwater drainage input^39–41^. In Białystok, rivers’ high concentrations of nitrate ions come from both limited groundwater supply and point pollution. In addition, urban rivers are often devoid of coastal vegetation, which results in little or no nitrate uptake^42^.

During drought, a decrease in iron ions concentration was observed. A similar relationship occurred in the Reedy river in South Carolina, where the most visible trend within the urban area of Greenville was the dramatic decrease in total iron concentrations during dry periods^43^. Hydrological drought also effects iron decrease in lowland rivers^40^. Comparison of the flow rate during drought with the characteristic flow in the Biała^15^ river with high probability indicates a low water level that is responsible for periodic hydrological drought (flow rate = 0.15 m^3^s^−1^). The recorded iron concentration was over 5 times lower than the average in the Biała river. Declining water tables influence aerobic and anaerobic microbial processes and concentrations of redox sensitive solutes like iron ions^9^.

The Biała river and its tributaries are still characterised by poor water quality, with elevated concentrations of nitrogen, phosphorus, chloride, iron and sodium^7,12,16^. The research by Ahn *et al*.^44^ shows that the increase of water pollution in urban areas may be multi-fold as compared to the quality of natural waters. The number of pollutants flowing into the water is correlated not only with the land use but, most of all, with impermeable surfaces like buildings, roads, sidewalks and parking lots^45^.

Our research shows high water quality variability along the Biała river. The rate of transport and deposition can differ broadly among stream reaches^46^. Many studies demonstrated that high ecological status of the river valley improves water quality^47,48^.

**Figure 8:**
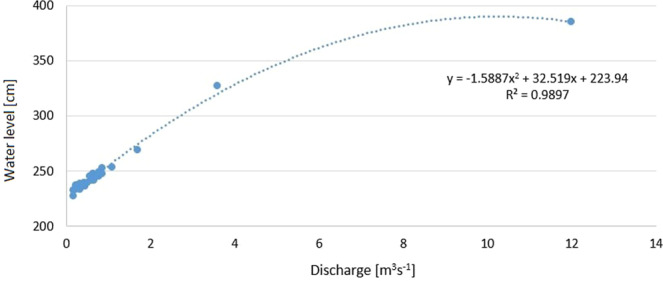
Rating curve of the Biała river in years 2014–2017.

Our research shows that the small river section with a high degree of naturalness is a buffer zone where the water quality could have improved during hydrological extremes. River restoration programs of the Biała river valley, conducted in the middle section of river, contributed to the increase of the ecological potential of river. The shape of the rating curve of the Biała river made based on data from 2014–2017, indicates the natural character of the river despite its location in the urbanised area (Fig. 8). This allowed testing of the hypothesis that a different response of water quality features in valleys with a varying degree of naturalness. A high degree of naturalness and increased water retention results in decreased concentration of ammonium, TP and DOC. Riparian buffer zones play an important role as nutrient pollution controls for rivers^49^. The described relationship is a very important observation in urban areas. It allows the necessity of revitalisation of water reservoirs to be stated. In urban catchments water components are highly variable and are characterised by non-stationarity at different scales. Given the interactions between anthropogenic sewage and drainage systems, designed for the rapid transfer of rainfall and water that has passed through the “natural” catchment areas, the processes taking place in these waters are completely different^34^.

## Conclusions

The quality of river waters in the area of Białystok is varied. The average EC value ranged widely from 300 µS cm^−1^ to 1,630 µS cm^−1^. Extreme hydro-meteorological phenomena affect surface water chemistry in small urban rivers. The consequence of flash floods is the dilution of the majority of surface water components, except for DOC and the most of phosphorous forms (DOP, PP, TP), for which inflow from the catchment causes an increase in their concentrations. Duration of precipitation effects concentrations of chemical parameters of river water. The storm rainfall results in an immediate change in water quality and maximum concentrations of carbon and phosphorus forms. After continuous rainfall, dilution of these compounds takes place, and the response is delayed in time with respect to the response to heavy rains. During hydrological droughts, the macronutrients and the mineral nitrogen forms are concentrated in rivers. The limited surface runoff causes a decrease in the concentration of carbon and phosphorus forms.

Even short sections of the natural river valleys with high ecological status work like buffer zones, and they result in improvement of water quality during hydrological extremes. This relationship confirms the need for urban water restoration, which will lead to increased biodiversity in urban areas and enable water use by urban residents.

## Data Availability

All data generated or analysed during this study are available via the Data repository of the University of Białystok. Requests for material should be made to the corresponding author.
